# Five-year outcomes and predictive factors of transforaminal full-endoscopic lumbar discectomy

**DOI:** 10.1097/MD.0000000000013454

**Published:** 2018-11-30

**Authors:** Yong Ahn, Uhn Lee, Woo-Kyung Kim, Han Joong Keum

**Affiliations:** a Department of Neurosurgery, Gil Medical Center, Gachon University College of Medicine, Incheon; b Department of Neurosurgery, Wooridul Spine Hospital, Seoul, South Korea.

**Keywords:** disc herniation, discectomy, full-endoscopic, lumbar, percutaneous, transforaminal

## Abstract

Although several studies have reported the effectiveness of transforaminal full-endoscopic lumbar discectomy (TELD), no cohort study on the long-term outcomes of TELD has been conducted. Thus, this study aimed to evaluate the long-term clinical outcomes of TELD and to determine the factors predicting favorable outcome.

Five-year longitudinal data of 204 consecutive patients who underwent TELD were collected. Outcomes were assessed using the visual analog scale (VAS) pain score, Oswestry disability index (ODI), patient satisfaction rating, and the modified Macnab criteria.

The mean VAS score for leg pain improved from 7.64 at the baseline to 1.71, 0.81, 0.90, and 0.99 at postoperative 6 weeks, 1 year, 2 years, and 5 years, respectively (*P* <.001). The mean ODI improved from 67.2% at the baseline to 15.7%, 8.5%, 9.4%, and 10.1% at postoperative 6 weeks, 1 year, 2 years, and 5 years, respectively (*P* <.001). The overall patient satisfaction rate was 94.1%. Based on the modified Macnab criteria, 83.8% of patients had excellent or good results. In this study, younger patients with intracanal disc herniation tended to have better outcomes than elderly patients with foraminal/far-lateral disc herniation (*P* <.05).

Transforaminal endoscopic lumbar discectomy offers favorable long-term outcomes with minimal tissue damage. Postoperative pain and functional status may change over time. Proper patient selection remains essential for the success of this minimally invasive procedure.

## 1 Introduction

Transforaminal full-endoscopic lumbar discectomy (TELD) has evolved to become one of the most minimally invasive spine surgeries. The basic concept of TELD is to directly approach the disc pathology through the foraminal window; this tends to result in decreased neuromuscular tissue damage compared to conventional techniques. Previous studies have demonstrated the effectiveness of full-endoscopic lumbar disc surgery via randomized controlled studies and meta-analyses.^[1–9]^ Although some spine surgeons criticize TELD for its relatively long learning curve and limited indications, the technique has seen several improvements. Initially, the procedure was performed as an indirect, intradiscal decompression under fluoroscopic guidance. However, with technical advancements in optics, surgical instruments, and access methods, the current TELD technique now involves a direct epidural fragmentectomy performed under high-quality endoscopic visualization.^[4,10–12]^ Despite these improvements, however, there are few relevant studies on the long-term results or the predictors of favorable outcomes for this technique. If any, the technique does not reflect the current endoscopic technique.^[13]^ Therefore, this study aimed to evaluate the long-term clinical outcomes and prognostic factors of the current TELD technique.

## 2 Materials and methods

### 2.1 Patients

This longitudinal cohort study included 229 patients with lumbar disc herniations who underwent TELD between January 2009 and December 2011. Patients were prospectively entered into the database and records were retrospectively reviewed. All TELD procedures were performed by 3 expert surgeons. Twenty-five patients (10.9%) were lost during the 5-year follow-up period. Thus, retrospective data were collected from the remaining 204 patients. This study was approved by the institutional ethical committee, and written informed consent was obtained from the patients. Patients with single-level symptomatic lumbar disc herniation despite more than 6 weeks of conservative treatment or those with acute disc herniation with progressive motor deficit were included in this study. Radicular pain with soft lumbar disc herniation was confirmed by both computed tomography (CT) and magnetic resonance imaging (MRI). The exclusion criteria included spinal stenosis, segmental instability, calcified disc herniation, massive disc herniation with cauda equina syndrome, and coexistent pathologic conditions, such as acute inflammation, infection, and tumor.

### 2.2 Surgical technique

TELD was performed under local anesthesia according to the standard transforaminal full-endoscopic technique.^[10,11,14]^ The surgical technique can be summarized as follows:

(1) fluoroscopic-guided percutaneous transforaminal approach,
(2) release of the annular anchorage and selective discectomy under direct endoscopic visualization, and
(3) confirmation of decompression and free mobilization of the nerve root.

**Figure 1:**
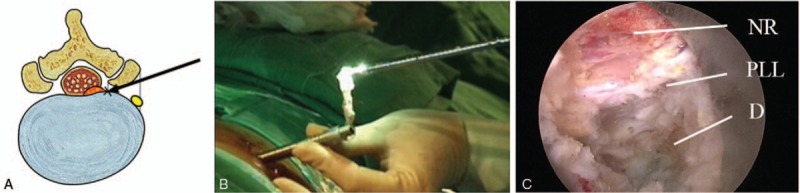
Schematic drawing and intraoperative views of the surgical technique. A. The basic principle of transforaminal approach is that the landing point should be as close to the target as possible and that the exiting nerve root should not be irritated. B. A large disc fragment is removed through a working sheath. C. At the final step, the anatomical details are well demonstrated including the decompressed NR, the PLL, and the maternal disc (D). NR = nerve root, PLL = posterior longitudinal ligament.

**Figure 2:**
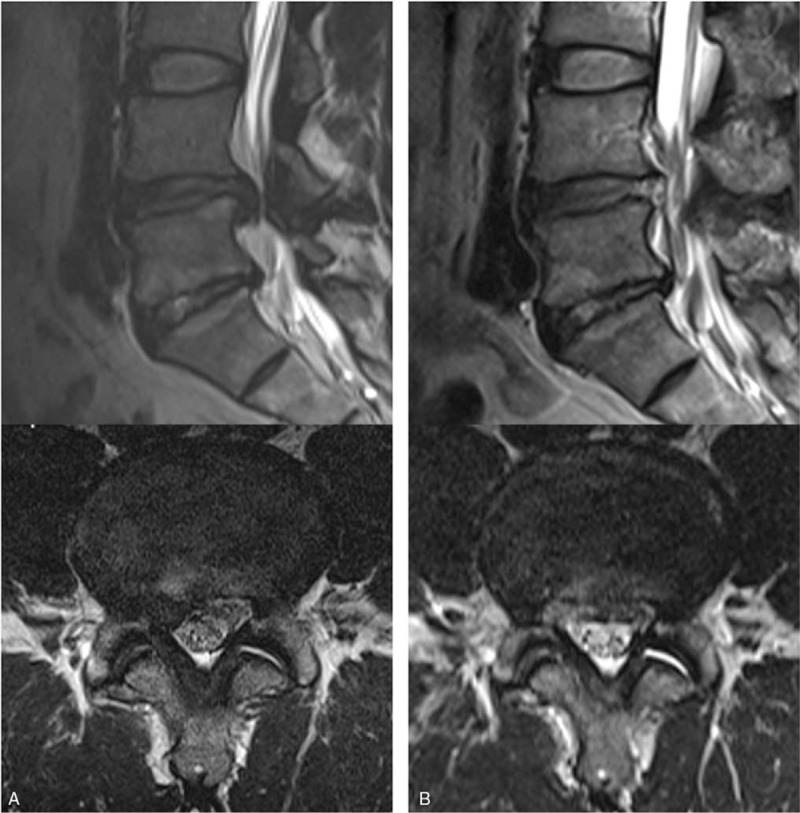
Illustrated case of a 44-year-old male patient with an excellent postoperative outcome. A. Preoperative MRI showing extruded disc herniation at the right L4-5 level. B. Postoperative MRI showing complete epidural decompression after selective removal of the herniated disc. MRI = magnetic resonance images.

Preoperatively, the patient is administered 0.05 mg/kg of midazolam intramuscularly and 0.8 μg/kg of fentanyl intravenously. Conscious sedation can be adjusted according to the patient's condition and the surgeon's need. In this technique, one of the most essential determinants of success is adequate transforaminal approach through the foraminal window. The 2 most important considerations are that the exiting nerve root should not be irritated and that the landing point should be as close to the target as possible. To protect the exiting nerve root, an approach that is slightly in the cranio-caudal direction is safer than a parallel trajectory to the disc space. For a correct landing, the approach angle and landing point should be adjusted according to the zone of disc herniation and the disc level. For central and subarticular disc herniation or lower lumbar disc herniation (L4–5 or L5-S1), a more shallow approach to the medial pedicular line is recommended. In contrast, for foraminal and far-lateral disc herniation or upper lumbar disc herniation (L3–4 and upper levels), a steeper approach angle to the medial pedicular line is recommended. At the L5-S1 level, in cases with a low iliac crest below the L5 pedicle, a routine transforaminal approach is usually possible. However, in cases with a high iliac crest above the L5 pedicle, a modified technique is required, with a more medial skin entry and resection of the superior articular process (e.g., foraminoplastic approach). Skin entry (typically 10–15 cm lateral) can be determined at the skin point between the tip of the spinous process and the posterior surface of the facet joint on the lateral fluoroscopic view. The needle is inserted into the target point through the foraminal window under fluoroscopic guidance, avoiding the exiting nerve root (Fig. 1A). Subsequently, a guide wire, serial dilators, and a final working sheath are placed in the epidural or intradiscal space. Second, the annular anchorage around the herniated fragment should be released by annulus scissors and a bipolar coagulator (Trigger-Flex Bipolar, Elliquence, Baldwin, New York). The released disc fragment can be selectively removed using various endoscopic forceps (Fig. 1B). This release-and-discectomy procedure is repeated until the nerve root and dural sac are decompressed. Complete herniotomy should be performed (i.e., removal of the whole iceberg) as remnants may cause symptom recurrence. Finally, the end point of the procedure is the free mobilization of the dural sac and nerve root (Fig. 1C). When the nerve root is adequately decompressed, the surgeon can confirm neural pulsation with the patient's pulse and cough. The patient should be observed for several hours for signs of any adverse events before discharge (Fig. 2).

### 2.3 Outcome evaluation and statistics

Patients’ outcome data were obtained during outpatient clinic follow-up visits using a patient-based outcome questionnaire or through telephone interviews. At each follow-up, patients completed a questionnaire that reflected their functional status and pain intensity. Patients’ back pain and radicular leg pain were assessed using the visual analog scale (VAS) pain score. Functional status was assessed using the Oswestry disability index (ODI).^[15]^ Clinical outcomes were assessed using the modified Macnab criteria^[10,16]^ and patient satisfaction rating.^[17,18]^ We classified the global outcomes into 4 groups according to the modified Macnab criteria: excellent (patients had no pain, had no mobility restriction, and could return to normal work), good (patients had occasional non-radicular pain, relief of the presenting symptoms, and the ability to return to modified work), fair (patients had some improved functional capacity, but were handicapped and/or unemployed), and poor (patients had no improvement, the objective symptoms had continued, or root involvement occurred; additional operative intervention was needed). Satisfaction rate was assessed before the patients’ discharge. Each patient answered the following question: “What is your level of satisfaction regarding the surgical procedure performed?” The patients chose 1 of 3 levels of satisfaction: very satisfied, satisfied, and unsatisfied.

Statistical analysis was performed by an independent statistician using SPSS 14.0K (SPSS, Inc., Chicago, IL). Each variable was subjected to univariate analysis to determine its relationship with the outcomes. For categorical variables, a Chi-square test or Fisher exact test was applied. Continuous variables were expressed as mean ± standard deviation and calculated using a Student *t* test. Multiple logistic regression analysis was also used to test the correlations among the different variables. A *P* value <.05 was considered statistically significant.

## 3 Results

### 3.1 Demographics and clinical outcomes

**Figure 3:**
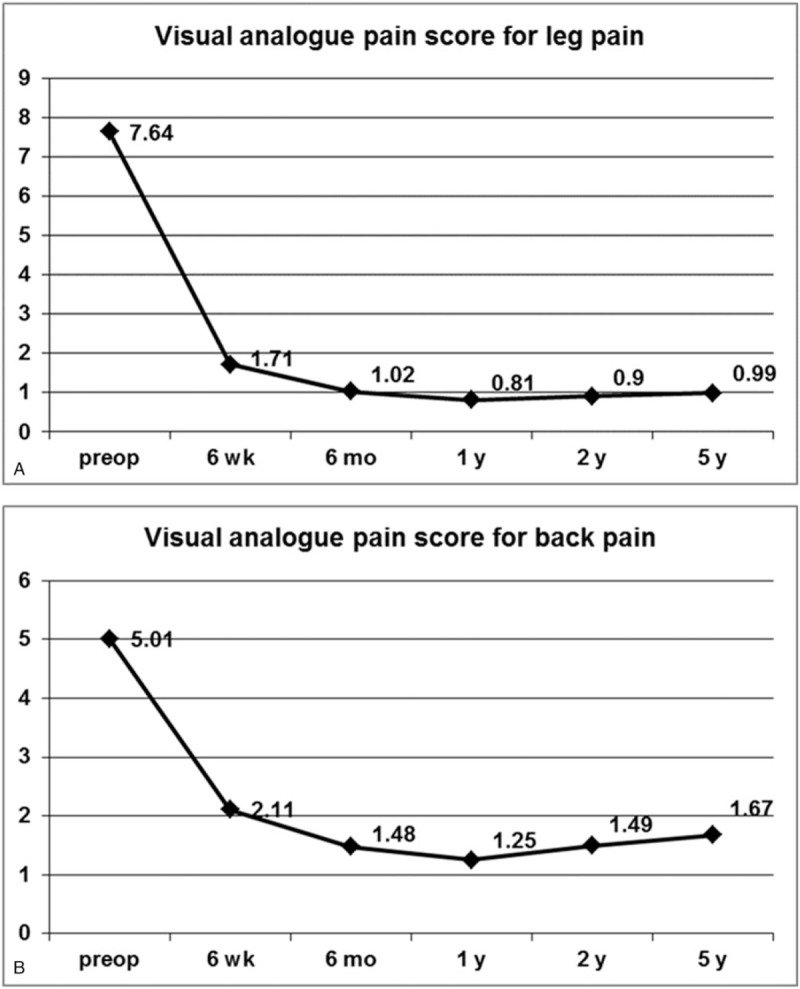
VAS preoperatively and at 6-weeks, 6-months, 1-year, 2-years, and 5-years postoperatively. A. VAS for radicular leg pain. B. VAS for back pain. VAS = visual analogue scale.

**Figure 4:**
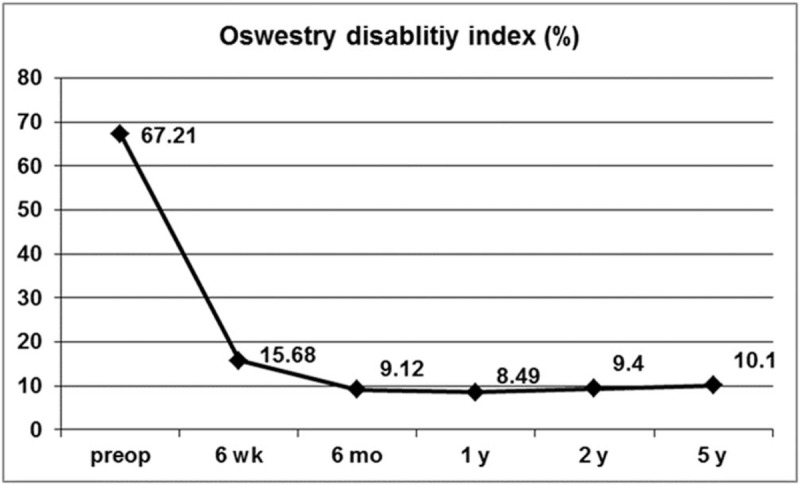
ODI preoperatively and at 6-weeks, 6-months, 1-year, 2-years, and 5-years postoperatively. ODI = Oswestry disability index.

**Figure 5:**
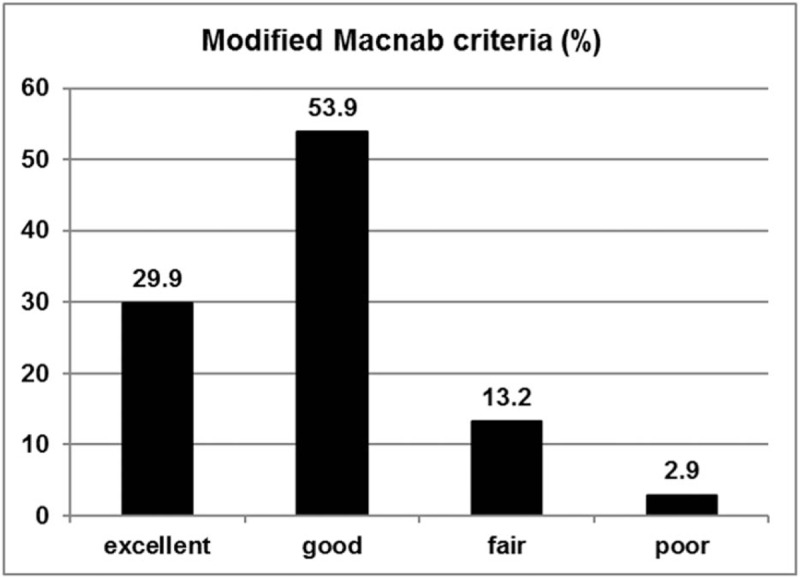
Global outcome based on the modified Macnab criteria: excellent in 61 (29.9%), good in 110 (53.9%), fair in 27 (13.5%), and poor in 6 (2.9%) patients.

This study included a total of 95 women (46.6%) and 109 men (53.4%) with a mean age of 32.9 years (range, 14 − 78 years). The mean operation time was 49.3 minutes (range, 25–100 min). The mean hospital stay was 1.84 ± 0.88 days. Patient demographics and neurologic findings are shown in Table 1. The mean time to return to work was 3.76 ± 1.21 weeks. Of the 204 patients, 174 patients (85.3%) could return to their ordinary work within 4 weeks. The mean preoperative VAS score for leg pain was 7.64 ± 1.35; postoperatively, the mean VAS score improved to 1.71 ± 1.43 and 0.99 ± 1.02 at postoperative 6 weeks and 5 years, respectively (*P* ≤.001; Fig. 3A). The mean VAS score for back pain was 5.01 ± 2.04 preoperatively and improved to 2.11 ± 1.00 and 1.67 ± 1.09 at postoperative 6 weeks and 5 years, respectively (*P* ≤.001; Fig. 3B). The mean preoperative ODI was 67.2 ± 13.7%, whereas the mean postoperative ODI was 15.7 ± 10.5% and 10.1 ± 11.9% at postoperative 6 weeks and 5 years, respectively (*P* ≤.001; Fig. 4). The patient satisfaction evaluation revealed that 51 (25%) patients were “very satisfied” and 141 (69.1%) patients were “satisfied” with their clinical results; the remaining 12 (5.9%) patients were “unsatisfied.” Thus, the overall patient satisfaction rate was 94.1%. At the final follow-up, patient outcomes were rated based on the modified Macnab criteria as follows: excellent in 61 (29.9%), good in 110 (53.9%), fair in 27 (13.2%), and poor in 6 (2.9%) patients. Therefore, excellent or good results were obtained in 83.8% (Fig. 5) of patients.

**Table 1:**
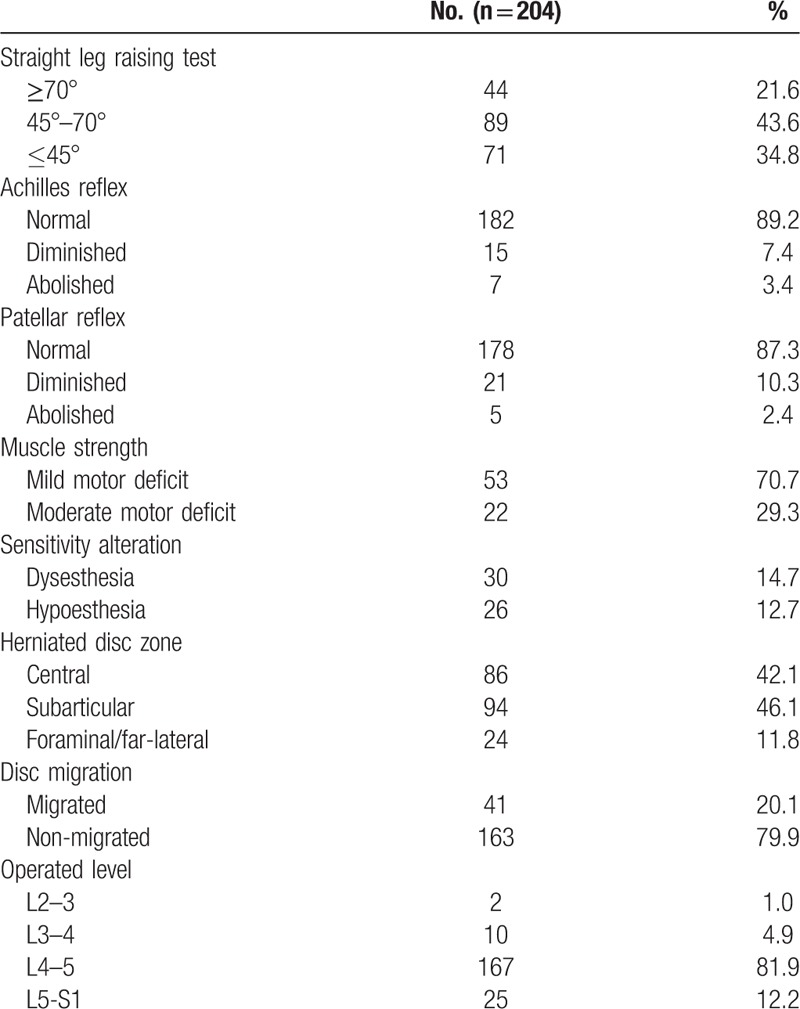
Patient demographics and preoperative information.

### 3.2 Complications and reoperation

**Figure 6:**
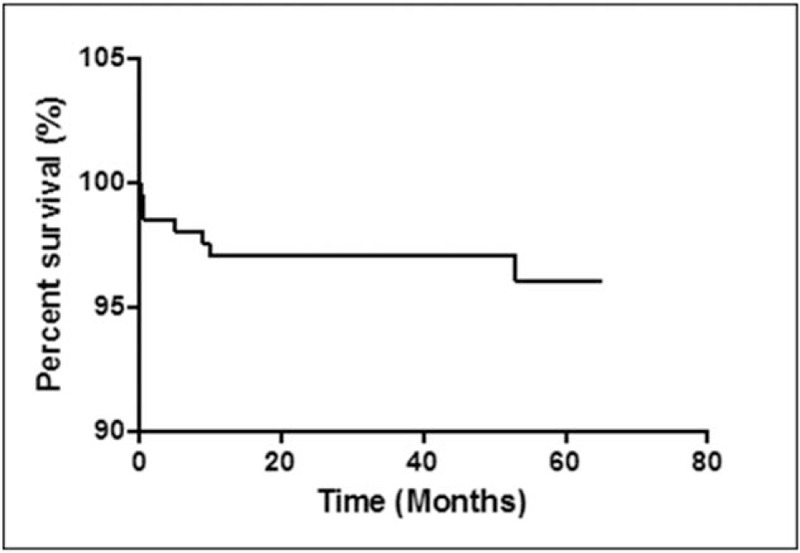
Survival curve for reoperations. Nine patients (4.4%) underwent subsequent open surgery for incomplete decompression or recurrent disc herniation. Seven reoperations were performed within 1 year; the remaining 2 reoperations were performed after 4 years.

Eight complications (3.9%) were reported, of which dysesthesia was the most common. Six patients experienced postoperative dysesthesia; 1 patient had a minor dural tear, which was intraoperatively sealed with gel foam and glue; and 1 patient had transient knee extension weakness, which improved within 3 months. Nine patients (4.4%) underwent subsequent open surgery due to incomplete decompression (4 patients) and recurrent disc herniation (5 patients). Of the 9 patients, 7 patients underwent open microdiscectomy for revision surgery and the remaining 2 patients underwent repeated TELD (Fig. 6). Five reoperations were performed within 6 weeks, 2 reoperations within 1 year, and 2 reoperations after 4 years.

### 3.3 Prognostic factors

Prognostic factors affecting the long-term outcomes were analyzed. For the preoperative variables, age at operation was related to the long-term outcomes. Patients <40 years old showed improved clinical outcomes (*P* <.001; Table 2). Another major predictive factor was the zone of disc herniation. An intracanal (central or subarticular) disc herniation demonstrated better outcomes than foraminal or far-lateral disc herniation (*P* <.001: Table 2). Other clinical and radiographic factors, including sex, height, weight, BMI, motor deficit, disc level, and presence of migrated disc herniation were not related to the long-term outcome. Forward stepwise multiple logistic regression showed that age (odds ratio [OR] = 3.748, *P* <.01) and zone of disc herniation (OR = 6.197, *P* <.001) were the most significant prognostic factors (Table 3). The predictive probability of successful outcome (excellent or good) for each patient was calculated by the following equation: *P* = exp Z/(1 + exp Z); Z = 1.321 X_1_ + 1.824 X_2_ − .601; X_1_ = age [0, 40 years or older; 1, younger than 40 years], X_2_ = zone of disc herniation [0, foraminal; 1, intracanal]. Table 4 shows the calculated predictive probabilities for different patient conditions, including age and zone of disc herniation. Younger age (<40 years) with intracanal disc herniation was estimated to lead to better outcome than older age or foraminal/far-lateral disc herniation.

**Table 2:**
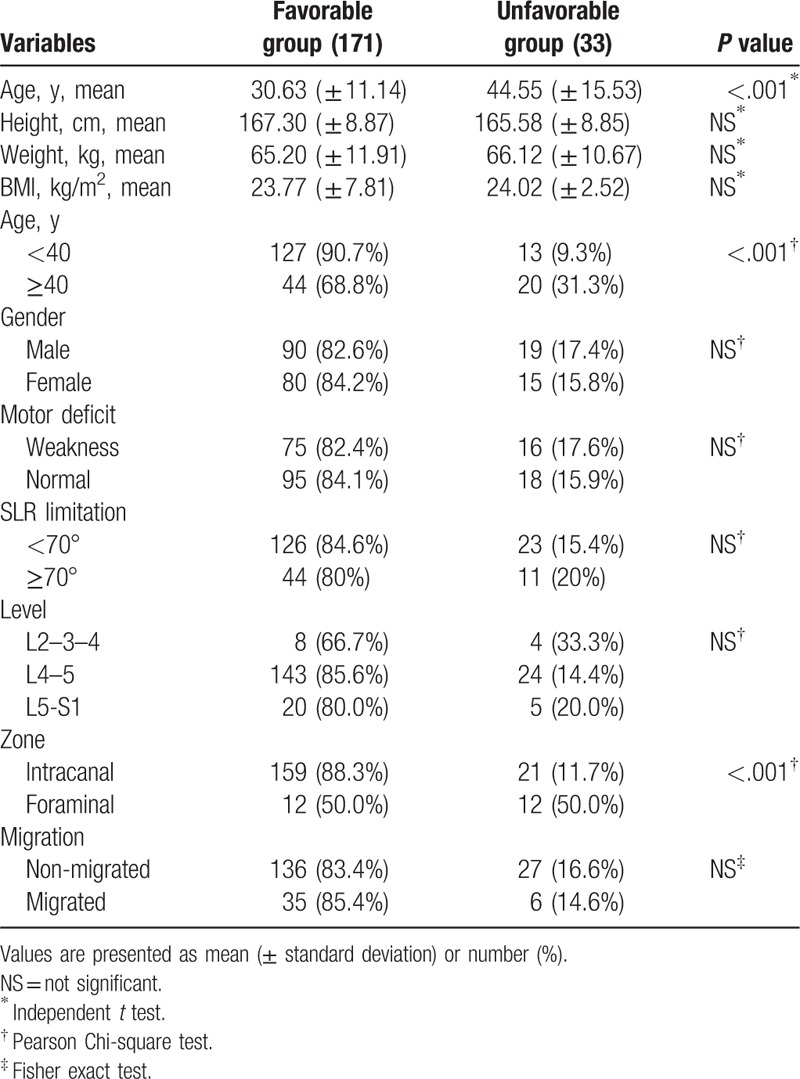
Statistical analysis of clinical and radiographic factors.

**Table 3:**
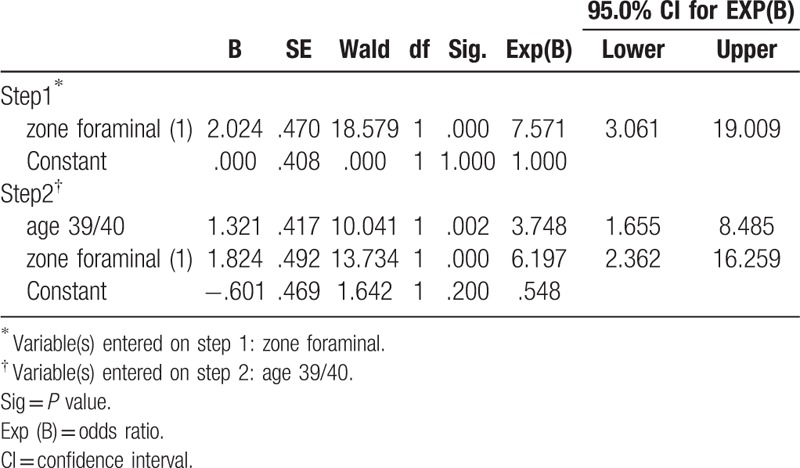
Binary logistic regression analysis.

**Table 4:**
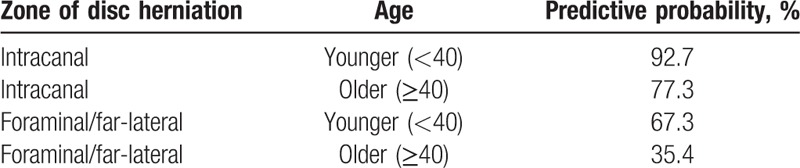
Predictive probability of favorable outcome.

## 4 Discussion

### 4.1 Long-term clinical outcomes and changes

This study demonstrated that patients who underwent TELD showed a statistically significant improvement in long-term postoperative pain scores and functional status. At 6 weeks, 1 year, 2 years, and 5 years postoperatively, the mean decrease in the VAS score for leg pain was 5.9 ± 1.8, 6.8 ± 1.6, 6.7 ± 1.6, and 6.6 ± 1.6, respectively; the mean decrease in the VAS score for back pain was 2.9 ± 1.8, 3.8 ± 2.0, 3.5 ± 2.2, and 3.3 ± 2.2, respectively; and the mean decrease in the ODI was 51.5 ± 13.0, 58.8 ± 13.6, 57.6 ± 14.1, and 56.9 ± 14.6, respectively. It has been suggested that a minimum 15-point reduction from the baseline ODI is clinically relevant.^[19]^ In this study, clinically significant improvement in ODI was observed in 192 patients (94.1%) at 6 weeks, 199 patients (97.5%) at 1 year, 197 patients (96.6%) at 2 years, and 194 patients (95.1%) at the final 5-year follow-up. According to the modified Macnab criteria, 83.8% of the patients in this study had successful outcomes (excellent or good), and 97.1% showed symptomatic improvement (excellent, good, or fair). Of the patients with symptomatic improvement, 29.9% had an excellent outcome, and the remaining 67.2% had definitive improvement in radiculopathy with mild back discomfort. We presumed that patients with excellent outcomes and no pain tended to report a level of satisfaction as “very satisfied” (25%), while those with symptomatic improvement and mild discomfort tended to answer as “satisfied” (69.1%). Taken together, these findings indicate that TELD is an effective technique capable of improving both symptoms and functional status in patients with nerve root compression due to a herniated disc.

Our data demonstrated a few interesting patterns over the 5-year follow-up period. First, pain scores and functional status steeply improved during the first 6 weeks. During the initial recovery period, some patients may experience transient discomfort or flare, in our study population, most symptomatic improvements become stable at postoperative 6 weeks. Then, outcome parameters steadily improved until postoperative 1 year. Thus, the pain reduction and functional status improvements were most notable at 1 year postoperatively. However, pain scores and disability indices gradually increased after 1 year. This phenomenon slightly progressed over the years; back pain was more prominent than radicular pain. Finally, our data showed that recurrent disc herniations could occur even after 4 years postoperatively. Casal-Moro et al also reported this trend after minimally invasive lumbar discectomy and concluded that the degenerative process continued over the years and could negatively affect postoperative pain scores and functional status in the long-term.^[19]^

### 4.2 Prognostic factors

Age was one of the major clinical factors affecting the long-term outcomes in our cohort. Patients <40 years old showed significantly improved pain score, functional status, and satisfaction rate. It is a generally accepted theory that younger patients have better results following lumbar disc surgery.^[14,20–22]^ This may be because younger patients tend to demonstrate a single-level disease and relatively healthy discs compared to older patients. The latter typically have multiple degenerated discs, which may also be related to degenerative changes of the disc after surgery. Moreover, older patients might have concurrent pathology, such as hypertrophic ligaments and facet joint arthropathy. However, these findings do not necessarily negate effectiveness of TELD when performed in older patients. Endoscopic surgery can be a suitable treatment option for older patients with concurrent medical diseases that are known to increase the risks of open surgery under general anesthesia.

**Figure 7:**
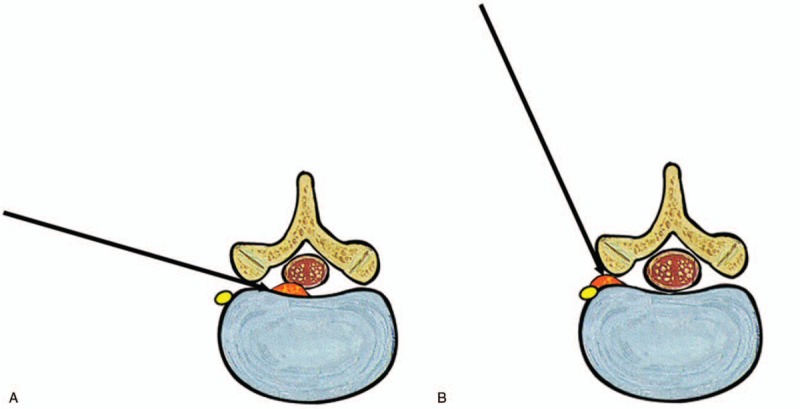
Schematic comparison of transforaminal approach according to the zone of disc herniation. A. For intracanal disc herniation, standard transforaminal approach can be performed avoiding the exiting nerve root and DRG. B. For foraminal or far-lateral disc herniation, steeper transforaminal approach is required and it may cause DRG irritation or postoperative dysesthesia. DRG = dorsal root ganglion.

Another significant prognostic factor determined in this study was the zone of disc herniation. Patients with foraminal or far-lateral disc herniation showed poorer outcomes compared to those with intracanal disc herniation, including central, and subarticular disc herniation (Fig. 7). There could be several reasons for this observation. First, previous studies have shown that irritation of the sensitive dorsal root ganglion (DRG) by foraminal or far-lateral disc herniation may cause postoperative residual symptoms.^[23,24]^ Second, a percutaneous transforaminal approach to the foraminal pathology may cause additional DRG irritation. This can be an inherent disadvantage of foraminal or transforaminal approach, especially for clinicians who are beginners of performing endoscopic spine surgery. Unlike the open posterior interlaminar approach, percutaneous access and docking to the narrowed foraminal disc with a blunt obturator and working sheath under fluoroscopic guidance is more likely to irritate the exiting nerve root. This irritation may result in incomplete decompression or postoperative dysesthesia. The incidence rate of postoperative dysesthesia after TELD is reported to range from 1.0% to 6.7% (average 2.5%).^[4,10,14,25–29]^ Both mechanical and thermal irritations may cause postoperative flare, with the latter resulting in more long-term negative effects. Once postoperative dysesthesia or flare occurs, regardless of the duration and degree, the negative effects on the patient's daily life could obscure any benefits of TELD.^[29,30]^ Moreover, negative effects of postoperative dysesthesia may persist during the long-term follow-up period. Therefore, preventing postoperative dysesthesia is vital for successful long-term outcomes, and learning to successfully do so could represent the last learning point for clinicians who wish to perform this technique.

### 4.3 Comparison of long-term outcomes of TELD with those of open lumbar discectomy

To date, open discectomy and microdiscectomy are considered the gold standard techniques for lumbar disc herniations.^[31,32]^ Previously published long-term satisfaction rates of the conventional technique range from 72% to 95%.^[33–46]^ As the indications differ for the conventional techniques and TELD, comparing satisfaction rates between the 2 may not always be possible. For example, the indication of open discectomy is broader than that for TELD. Full-endoscopic discectomy is considered effective for soft disc herniation, and concurrent spinal stenosis or calcified disc herniation is not usually indicated for TELD. However, some randomized trials have compared the results of TELD and of open discectomy for soft disc herniation^[1,2,4,5]^; these studies showed that the effectiveness of TELD was comparable to that of the conventional technique, with the typical benefits of a minimally invasive technique. For long-term follow-up results, in terms of satisfaction and revision rate, TELD outcomes in the present study were comparable to those of conventional open lumbar discectomy in published series (Table 5).

**Table 5:**
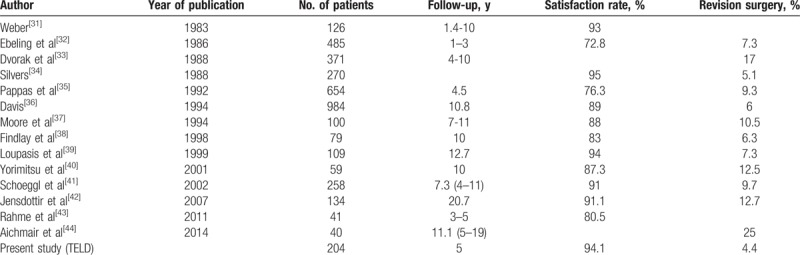
Comparison of the long-term results with conventional open lumbar discectomy (OLD).

### 4.4 Limitations of the study

Although this cohort study was performed according to standard protocols and included a large number of patients, some limitations exist. First, selection bias in the patients’ enrollment was possible. The operating surgeons may have chosen younger patients or preferred patients with disease at L3–4 or L4–5 level for endoscopic surgery over those with disease at L5-S1. Second, no control patients, that is, those who treated with open lumbar discectomy or microdiscectomy, were included in this study. However, the main goal of this study was to evaluate pain and functional status changes over the years after TELD. Indirect comparisons can be made through literature review, and comparison between the long-term effectiveness of endoscopic surgery and conventional open surgery will be the topic of our future study.

## 5 Conclusion

TELD appears to show long-term effectiveness for treating soft lumbar disc herniation, resulting in minimal tissue damage and a reduced disability period. In this study, postoperative pain and functional status changed over time during the 5-year follow-up period. Prognosis was significantly better in younger patients (<40 years) with intracanal disc herniation compared to older patients or those with foraminal/far-lateral disc herniation.

## Acknowledgments

The authors would like to thank Jin Ah Kim, Jae Min Son, and Sang Ho Lee for their support and assistance with this study.

## Author contributions

**Conceptualization:** Yong Ahn, Uhn Lee.

**Data curation:** Yong Ahn, Han Joong Keum.

**Formal analysis:** Yong Ahn, Uhn Lee.

**Funding acquisition:** Yong Ahn.

**Investigation:** Yong Ahn.

**Methodology:** Yong Ahn, Woo-Kyung Kim.

**Project administration:** Yong Ahn.

**Resources:** Uhn Lee, Han Joong Keum.

**Software:** Uhn Lee.

**Supervision:** Uhn Lee, Woo-Kyung Kim.

**Validation:** Yong Ahn, Uhn Lee, Woo-Kyung Kim, Han Joong Keum.

**Visualization:** Yong Ahn.

**Writing – original draft:** Yong Ahn.

**Writing – review & editing:** Yong Ahn, Uhn Lee, Woo-Kyung Kim, Han Joong Keum.
